# Generation of the Sotos syndrome deletion in mice

**DOI:** 10.1007/s00335-012-9416-0

**Published:** 2012-08-29

**Authors:** Anna M. Migdalska, Louise van der Weyden, Ozama Ismail, The Sanger Mouse Genetics Project, Alistair G. Rust, Mamunur Rashid, Jacqueline K. White, Gabriela Sánchez-Andrade, James R. Lupski, Darren W. Logan, Mark J. Arends, David J. Adams

**Affiliations:** 1 Experimental Cancer Genetics, Wellcome Trust Sanger Institute, Wellcome Trust Genome Campus, Hinxton, Cambridge, CB10 1HH UK; 2 Mouse Genetics Project, Wellcome Trust Sanger Institute, Wellcome Trust Genome Campus, Hinxton, Cambridge, CB10 1HH UK; 3 Genetics of Instinctive Behaviour, Wellcome Trust Sanger Institute, Wellcome Trust Genome Campus, Hinxton, Cambridge, CB10 1HH UK; 4 Department of Molecular and Human Genetics, Baylor College of Medicine, Houston, TX 77030 USA; 5 Department of Pathology, Addenbrooke’s Hospital, University of Cambridge, Cambridge, CB2 0QQ UK

## Abstract

Haploinsufficiency of the human 5q35 region spanning the *NSD1* gene results in a rare genomic disorder known as Sotos syndrome (Sotos), with patients displaying a variety of clinical features, including pre- and postnatal overgrowth, intellectual disability, and urinary/renal abnormalities. We used chromosome engineering to generate a segmental monosomy, i.e., mice carrying a heterozygous 1.5-Mb deletion of 36 genes on mouse chromosome 13 (*4732471D19Rik-B4galt7*), syntenic with 5q35.2–q35.3 in humans (*Df*(*13*)*Ms2Dja* ^*+/−*^ mice). Surprisingly *Df(13)Ms2Dja* ^*+/−*^ mice were significantly smaller for their gestational age and also showed decreased postnatal growth, in contrast to Sotos patients. *Df(13)Ms2Dja* ^*+/−*^ mice did, however, display deficits in long-term memory retention and dilation of the pelvicalyceal system, which in part may model the learning difficulties and renal abnormalities observed in Sotos patients. Thus, haploinsufficiency of genes within the mouse *4732471D19Rik*–*B4galt7* deletion interval play important roles in growth, memory retention, and the development of the renal pelvicalyceal system.

**Electronic supplementary material:**

The online version of this article (doi:10.1007/s00335-012-9416-0) contains supplementary material, which is available to authorized users.

## Introduction

Sotos syndrome (Sotos; MIM# 117550) is an autosomal dominant, multiple-anomaly syndrome characterized by overgrowth, a distinctive craniofacial appearance, advanced bone age, and variable learning disabilities. However, there is significant clinical heterogeneity in Sotos syndrome, with some affected individuals also showing frequent ear and chest infections, cardiac and urinary/renal defects, seizures, scoliosis, and behavioural problems (Tatton-Brown et al. 1993). The diagnosis of Sotos syndrome relied solely on clinical criteria until haploinsufficiency of the *NSD1* gene (encoding a histone methyltransferase implicated in chromatin regulation) was identified as causative (Kurotaki et al. 2002). Subsequent analysis of patients clinically diagnosed with Sotos showed that haploinsufficiency of *NSD1* due to intragenic *NSD1* mutations, partial *NSD1* deletions, or chromosomal microdeletions spanning the 5q35 region encompassing the entire *NSD1* gene accounted for more than 90 % of cases (with the prevalence of intragenic *NSD1* mutations and 5q35 microdeletions encompassing the *NSD1* gene depending greatly on ethnic origin) (Baujat et al. 2004; Tatton-Brown and Rahman 2007; Tatton-Brown et al. 2005a, b). It has also been proposed that *GPC3* mutations or 11p15 abnormalities or both may be responsible for some Sotos cases without *NSD1* abnormalities (Baujat et al. 2004; Li et al. 2001; Tatton-Brown and Rahman 2007).

The major clinical features of Sotos, including overgrowth, facial abnormalities, and intellectual disabilities, are diagnosed in Sotos patients with intragenic *NSD1* mutations and in Sotos individuals carrying 5q35 microdeletions (Tatton-Brown et al. 2005a, b). However, in contrast to Sotos individuals with *NSD1* mutations, Sotos patients with 5q35 microdeletions tend to show less pronounced overgrowth but more profound intellectual disability (Kurotaki et al. 2003; Nagai et al. 2003; Saugier-Veber et al. 2007; Tatton-Brown et al. 2005a), and several studies have reported an increased frequency of cardiovascular and urinary/renal abnormalities in 5q35 microdeletion Sotos patients (Kurotaki et al. 2003; Nagai et al. 2003; Saugier-Veber et al. 2007). Thus, it is possible that genes other than the *NSD1* gene could be dosage-sensitive and therefore responsible for the extended variability and degree of severity of phenotypes observed in Sotos patients who carry 5q35 microdeletions. Furthermore, selected patients with Sotos can have low laboratory values of the factor XII blood-clotting protein, a phenotype thought to result from the hemizygous deletion of 5q35, unmasking a functional single nucleotide variant (SNV) on the remaining nondeleted allele of the *FXII* gene within the common Sotos deletion interval (Kurotaki et al. 2005). The latter genetic mechanism has recently also been shown to be responsible for TAR (thrombocytopenia absent radius) syndrome where 1q21.1 deletions appear to unmask functional variants associated with the *RBM8a* gene (Albers et al. 2012).

The mouse orthologue of the *NSD1* gene (*Nsd1*) is highly conserved (83 % homology at amino acid level) (Kurotaki et al. 2001); however, heterozygous *Nsd1* mice do not display any gross phenotypic abnormalities (Rayasam et al. 2003). Similarly, mice carrying knockout mutations of other genes within the most frequently detected human Sotos 5q35 microdeletion interval have failed to display dominant phenotypes, suggesting that *Sncb*, *Unc5a*, *Fgfr4*, *Mxd3*, *Rgs14*, *Slc34a1*, *F12*, *Grk6*, *Dbn1*, and *Dok3* are not dosage-sensitive and, therefore, at least individually are not likely to be responsible for the extended variability and degree of severity of clinical features observed in 5q35 microdeletion Sotos patients (Mouse Genome Informatics; http://www.informatics.jax.org). In order to allow investigation of the contribution of the 36 genes at the distal end of human chromosome 5 to the development of clinical features commonly diagnosed in Sotos patients, we used chromosome engineering to generate a new genetic mouse model of Sotos syndrome, *Df(13)Ms2Dja* ^*+/−*^, carrying a deletion syntenic to the human chromosome 5q35.2–q35.3 region.

## Materials and methods

### Gene targeting in ES cells and generation of deletion mice

The 5′ *Hprt* MICER targeting vector MHPN55m07 (Adams et al. 2004) was linearized with *Kpn*I and electroporated into E14Tg2a embryonic stem (ES) cells (129P2Ola), which were selected in G418 as described previously (Ramirez-Solis et al. 1995). Southern blotting was performed on *Bst*EII-digested ES cell genomic DNA (gDNA) using a probe amplified from E14Tg2a gDNA (5′-GTC TGT TGT TAA AAG CTA AAA CCT TAG A-3′ and 5′-TGA GCT ACA GTT TGG TTC TGG TGG ATA AAC-3′) to identify correctly targeted clones. The 3′ *Hprt* MICER targeting vector MHPP265c24 (Adams et al. 2004) was linearized with *Nco*I and electroporated into MHPN55m07-targeted E14Tg2a ES cells, which were then selected in puromycin. Southern blotting was performed on *Spe*I-digested gDNA using a probe amplified from E14Tg2a gDNA (5′-CAG TAA TAT AGT AGA AGC ATG GTC CAT-3′ and 5′-ATG ATA CTG AAC ACA GAC AAC AGA GGC TGC T-3′). Double-targeted ES cell clones were electroporated with a Cre-expression vector and selected in hypoxanthine, aminopterin, and thymidine (HAT) medium, as described previously (Ramirez-Solis et al. 1995), to identify whether the 5′ *Hprt* and 3′ *Hprt* vectors were targeted in *cis* or in *trans*. Clones of ES cells that had undergone *cis* recombination were identified and confirmed by PCR (5′-AAG GGT GTT TAT TCC CCA TGG ACT AAT TAT G-3′ and 5′-CCT TCA TCA CAT CTC GAG CAA GAC GTT CAG-3′; presence of a 1.7-kb band confirmed the *cis* orientation). The deletion allele was designated *Df(13)Ms2Dja*. ES cell clones carrying the conditional deletion (prior to electroporation with Cre) were injected into C57BL/6-*Tyr* ^*c-Brd*^ blastocysts and transmitted through the germline. F1 mice carrying the conditional deletion were bred with CMV-Cre mice (Su et al. 2002) to generate heterozygous *Df(13)Ms2Dja* mice (*Df(13)Ms2Dja* ^*+/−*^). These deficiency mice, *Df(13)Ms2Dja* ^*+/−*^, were backcrossed to C57BL/6J for three to four generations and maintained on a mixed C57BL/6J-129P2Ola background. All mice were housed and experimental procedures were carried out in accordance with UK Home Office guidelines.

### Comparative genomic hybridization (CGH)

CGH analysis was performed on the Agilent 244A array using tail DNA. The data were processed and analysed in R (R Foundation for Statistical Computing, Vienna, Austria).

### Fluorescent in situ hybridization (FISH)

Chromosome spreads of activated splenocytes and ES cells were generated and labelled as described previously (Migdalska et al. 2012; Robertson 1987). Mouse BAC clones (http://bacpac.chori.org/mmouse24.htm) were located inside (RP23-99C7) or outside (RP24-204D5) the deletion region and labelled with DIG-dUTP or biotin-dUTP, respectively.

### Histology

Tissues were fixed in 10 % neutral buffered formalin for 24 h and embedded in paraffin; then 5-mm sections were cut and stained with haematoxylin and eosin.

### Phenotypic assays

#### Body weight and length analysis

Embryos were weighed at day 15.5 of gestation (E15.5), and mice were weighed at 1 day, or 10, 28, or 52 weeks of age. The body length measurements were taken at 10 and 28 weeks of age.

#### Dual-energy X-ray absorptiometry (DEXA)

Mice at 10 or 28 weeks of age were analysed on a PIXImus II Densitometer (GE Medical Systems, Buckingshire, UK) as described previously (Migdalska et al. 2012). Data were statistically analysed using the two-tailed Student’s *t*-test.

#### X-ray imaging

Mice at 28 and 52 weeks of age were euthanized and assessed using the MX20 Faxitron system (Faxitron Bioptics, LLC, Lincolnshire, IL). Two lateral X-ray images were acquired: a whole-body image and a lower abdominal image. The latter images were analysed to check for the presence of stones in the kidneys.

#### Behavioural tests

Mice were group-housed from weaning age (3 weeks) and behavioural tests performed at 22–28 weeks of age. All mice were prehandled for 1–2 min every day for 4 days prior to testing for habituation. Two tests were performed, specifically a social recognition test and an olfactory/vomeronasal function test, both as previously described (Migdalska et al. 2012).

## Results

### Generation of *Df(13)Ms2Dja*^*+/−*^ mice carrying a 1.5-Mb deletion of the *4732471D19Rik–B4galt7* region

**Fig. 1:**
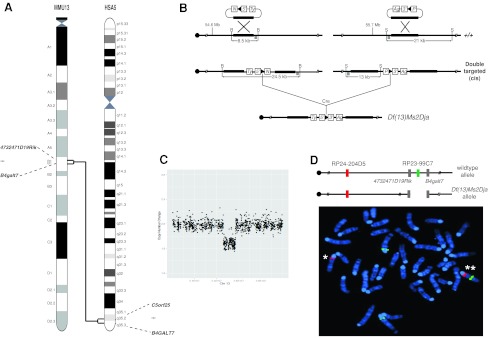
Generation of a 1.5-Mb deletion between the *4732471D19Rik* and *B4galt7* loci. **a** Schematic representation of HSA5 and the syntenic region on MMU13 with the end points of the syntenic regions indicated (*4732471D19Rik* and *B4galt7*). Genes that map to the human 5q35.2–q35.3 region (NCBI build GRChr37) and the B1 band on MMU13 (NCBI build m37) are listed in Supplementary Table 1. **b** The targeting vectors used to generate the 1.5-Mb deletion between the *4732471D19Rik* and *B4galt7* loci contained a *loxP* site (*arrowhead*), a selectable antibiotic resistance gene [*N* (neomycin) or *P* (puromycin)], a coat-colour marker (*Ty* or *Ag*), and part of the *Hprt* gene (5′ or 3′), which were targeted as indicated (B, *Bst*EII; S, *Spe*I; P, puromycin; N, neomycin; Ty, Tyrosinase; Ag, Agouti). **c** Comparative genomic hybridization of the Sotos deletion interval. **d** Interphase FISH analysis with BAC probes that map either inside (*green*) or outside of (*red*) the deletion interval. Chromosomes from the ES cells double-targeted in *cis* (*Df(13)Ms2Dja* ^*+/−*^) showed a* red* signal indicating deletion of the *4732471D19Rik-B4galt7* interval (*), while the wild-type chromosome showed* red* and* green* signals (**). There are two additional* green* centromeric signals present due to nonspecific binding of the probe

The *4732471D19Rik* and *B4galt7* genes are located at the proximal and distal ends of a 1.5-Mb region in the B1 band of mouse chromosome 13 (MMU13), which is syntenic to the human cytogenetic region 5q35.2–q35.3 and the genomic interval Chr 5: 175–177 Mb (Fig. 1a). This region on human chromosome 5 (HSA5) contains 36 genes (NCBI build GRChr37), all of which have orthologous counterparts found on the syntenic region of MMU13 (NCBI build m37) (Supplementary Table 1). We used chromosomal engineering to generate the deletion (Zheng et al. 1999), whereby E14Tg2a ES cells were sequentially electroporated with targeting vectors containing a portion of the *Hprt* selection cassette (5′ or 3′ *Hprt*), a *loxP* site, and a coat-colour marker (Agouti or Tyrosinase). The targeting vector containing the 5′ *Hprt* cassette (MICER clone: MHPN55m07) (Adams et al. 2004) was inserted proximal to *4732471D19Rik* and the targeting vector containing the 3′ *Hprt* cassette (MICER clone: MHPP265c24) (Adams et al. 2004) was inserted distal to *B4galt7* (Fig. 1b). The correct insertion of both targeting vectors was confirmed by Southern blot analysis on *Bst*EII- or *Spe*I-digested genomic DNA extracted from ES clones using external probes. Double-targeted ES cell clones were electroporated with a Cre-expression vector and selected in medium containing hypoxanthine, aminopterin, and thymidine (HAT) to identify lines carrying chromosomal deletions generated via *loxP-*mediated recombination. Clones in which the vectors were targeted in *cis* generated hundreds of colonies, while those targeted in *trans* generated virtually no colonies. The deletion allele was designated *Df(13)Ms2Dja*, and deletion of the interval between the targeting vectors was confirmed by fluorescence in situ hybridisation (FISH) on *Hprt*-resistant ES clones (Fig. 1d). *Cis*-targeted conditional ES cell clones were injected and the double-targeted un-recombined (floxed) chromosome was transmitted through the germline. The resulting F1 mice were bred with a Cre deleter strain (CMV-Cre mice) to generate *Df(13)Ms2Dja* ^*+/−*^ mice. *Df(13)Ms2Dja* ^*+/−*^ mice were backcrossed to C57BL/6J for three to four generations and maintained on a mixed C57BL/6J-129P2/Ola background. FISH analysis was performed on splenocytes, as described above, to revalidate the allele before experiments were performed using *Df(13)Ms2Dja* ^*+/−*^ mice. CGH analysis of tail DNA also confirmed the establishment of the *Df(13)Ms2Dja* deletion in mice (Fig. 1c).

### *Df(13)Ms2Dja*^*+/−*^ embryos are small for their gestational age

**Fig. 2:**
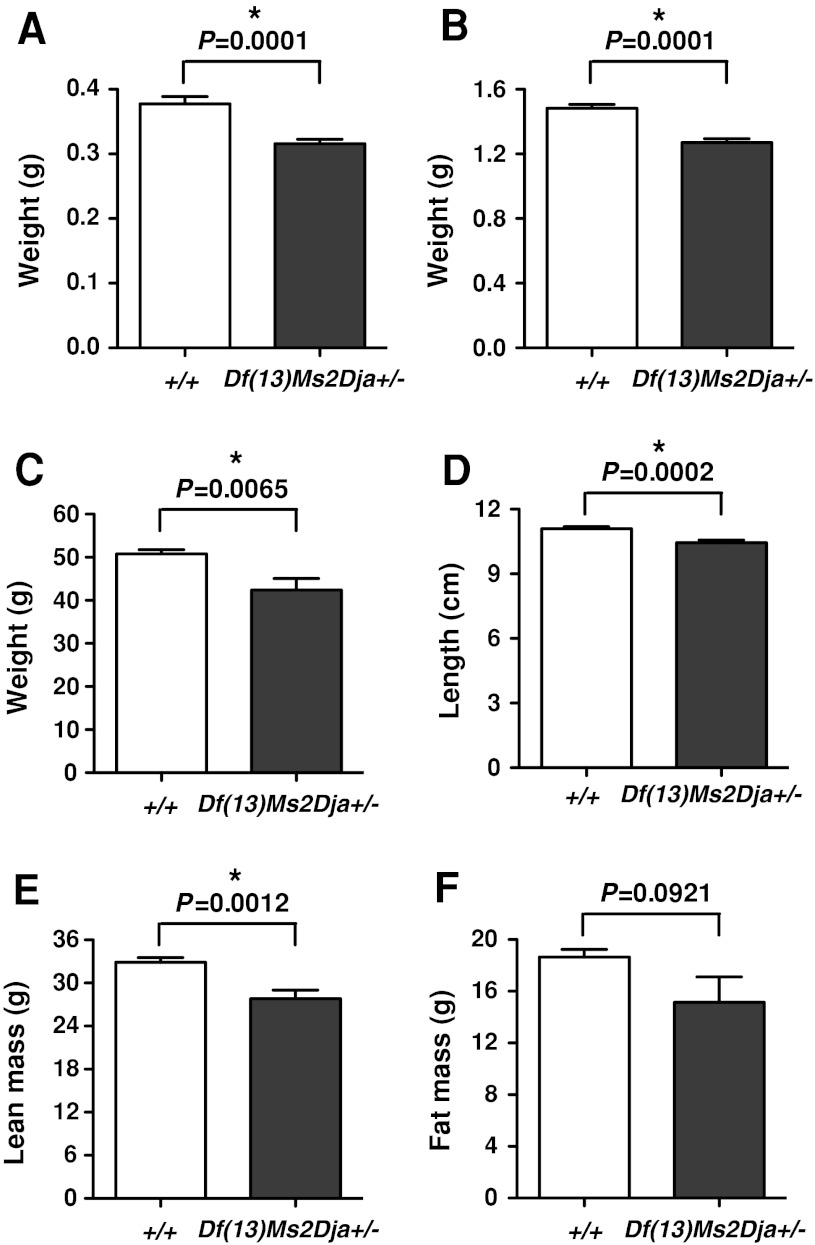
Phenotypic analysis of *Df(13)Ms2Dja* ^*+/−*^ mice at various time points. **a** Body weight measurements in control (*+/+*, *n* = 12) and deficiency (*Df(13)Ms2Dja* ^*+/−*^, *n* = 12) littermates at embryonic day 15.5. **b** Body weight measurements in control (*+/+*, *n* = 25) and deficiency (*Df(13)Ms2Dja* ^*+/−*^, *n* = 21) littermates at 1 day of age. **c**, **d** Body weight and length measurements in control (*+/+*, *n* = 12) and deficiency (*Df(13)Ms2Dja* ^*+/−*^, *n* = 11) littermates at 28 weeks of age. **e**, **f** DEXA analysis of lean mass and fat mass in control (*+/+*, *n* = 12) and deficiency (*Df(13)Ms2Dja* ^*+/−*^, *n* = 11) littermates at 28 weeks of age. All data were statistically analysed using the two-tailed Student’s *t*-test, and the *asterisk* indicates statistical significance. The *error bars* represent the standard deviation of the measurements

To determine whether clinical features of Sotos patients could be observed in *Df(13)Ms2Dja* ^*+/−*^ mice, we initiated our studies during embryonic development. At embryonic day 15.5 (E15.5), *Df(13)Ms2Dja* ^*+/−*^ embryos showed significantly reduced growth (as measured by reduced body weight) for their gestational age when compared with wild-type littermate embryos (Fig. 2a). It is unlikely that this difference is due to the genetic background of these mice (mixed C57–129), as the same phenotype was observed after a one-generation backcross to different strains (specifically, BALB/cJ, CBA/J, and 129/S5SvEv; Supplementary Fig. 1). Reduced growth of *Df(13)Ms2Dja* ^*+/−*^ animals was also evident upon examination of 1-day-old pups compared with wild-type littermates (Fig. 2b). These observations are contrary to human Sotos foetuses and newborns that typically show an overt overgrowth phenotype and suggest differences in the role that *NSD1* and *Nsd1* play in embryonic and postnatal development in human and mouse, respectively.

### Adult *Df(13)Ms2Dja*^*+/−*^ mice are smaller and lighter than their littermates

*Df(13)Ms2Dja* ^*+/−*^ mice fed on a normal-fat diet were lighter and shorter than their wild-type littermates at 28 weeks (males, Fig. 2c, d; females, data not shown). Detailed analysis of the body composition of these mice revealed a statistically significant decrease in the lean mass, but not fat mass, of *Df(13)Ms2Dja* ^*+/−*^ mice (males, Fig. 2e, f; females, data not shown).

In addition, to determine whether clinical features of Sotos patients, including macrocephaly, facial abnormalities, hypotonia, seizures, and/or scoliosis, could be observed in adult *Df(13)Ms2Dja* ^*+/−*^ mice, they were subjected to dysmorphology and X-ray analysis. However, *Df(13)Ms2Dja* ^*+/−*^ mice were viable, fertile, and did not show any overt skeletal or craniofacial phenotypes (data not shown).

### Behavioural phenotyping of adult *Df(13)Ms2Dja*^*+/−*^ mice

**Fig. 3:**
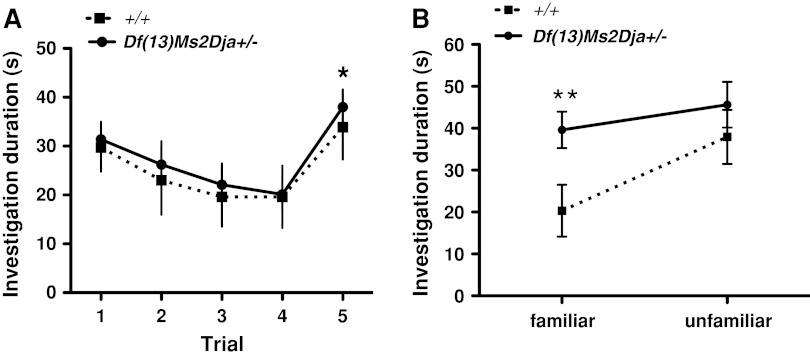
Learning impairment in *Df(13)Ms2Dja* ^*+/−*^ mice. **a** Habituation-dishabituation test. Both deficiency (*Df(13)Ms2Dja* ^*+/*−^, *n* = 9) and control (*+/+*, *n* = 8) mice recognized two different stimulus animals, as shown by a decline in the investigation time over trials 1–4 when they were repetitively presented the same stimulus animal (mouse A), and an increase in the investigation time on trial 5 when they were presented with a novel stimulus animal (mouse B) (trial 4 vs. trial 5, *P* < 0.0001, post-hoc analysis after two-way ANOVA). **b** Discrimination test (performed 24 h after the habituation-dishabituation test). *Df(13)Ms2Dja* ^*+/−*^ mice spent significantly more time investigating the familiar stimulus mouse (mouse A) than did control mice (*P* < 0.05, two-tailed Student’s *t*-test), suggesting that *Df(13)Ms2Dja* ^*+/−*^ mice were less able to distinguish a familiar from an unfamiliar animal (mouse C). The *error bars* represent the standard error of the mean and the *asterisks* indicate statistical significance. Four animals (one deficiency and three wild types) were withdrawn from the experiment because of their low investigation times (less than 10 s on trial 1)

To identify whether the learning disability of Sotos patients could be observed in adult *Df(13)Ms2Dja* ^*+/−*^ mice, the mice were subjected to two olfactory discrimination tests (social recognition paradigms with a long-term memory component) (Engelmann et al. 2011). A “habituation-dishabituation” test showed that deficiency mice were similar to wild-type littermates in their ability to recognize two different stimulus animals, as shown by the decline in the investigation time over trials 1–4 when they were repetitively presented the same animal (mouse A), and an increase in the investigation time on trial 5 when they were presented with a novel stimulus animal (mouse B) (Fig. 3a; two-way ANOVA with repeated measures for Trial, *P* < 0.0001, and post-hoc test trial 4 vs. trial 5, *P* < 0.0001). Both groups of mice had similar initial levels of investigation and spent increasingly less amounts of time investigating the repeatedly presented stimulus animal, suggesting normal levels of anxiety and social olfactory-mediated interaction for *Df(13)Ms2Dja* ^*+/−*^ animals. However, the “discrimination test” showed that deficiency mice were less capable of distinguishing the familiar stimulus animal (mouse A) 24 h later (two-way ANOVA with repeated measures, effect of Genotype, *P* = 0.045). They spent a significantly longer time investigating the familiar mouse than did the wild-type littermate controls (Fig. 3b; Bonferroni post-hoc test wild-type vs. *Df(13)Ms2Dja* ^*+/−*^ mice, *P* < 0.05). To confirm that this deficit was cognitive and not simply a consequence of diminished olfactory/vomeronasal capacity in *Df(13)Ms2Dja* ^*+/−*^ mice, we ascertained that they could recognize and discriminate between signature odours in the absence of other sensory cues with ability equal to that of their wild-type littermates (Supplementary Fig. 2). Thus, *Df(13)Ms2Dja* ^*+/−*^ mice show a degree of learning disability.

### Histopathological analysis of *Df(13)Ms2Dja*^*+/−*^ mice reveals dilation of the renal pelvicalyceal system

**Fig. 4:**
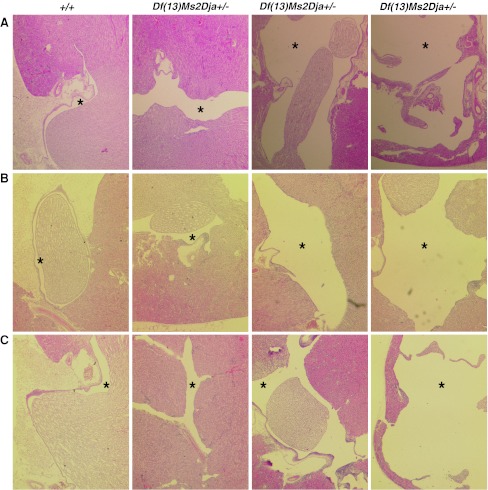
Dilation of the renal pelvicalyceal system in *Df(13)Ms2Dja* ^*+/−*^ mice. Haematoxylin and eosin-stained kidney sections from control (*+/+*) and deficiency (*Df(13)Ms2Dja* ^*+/−*^) mice at **a** 10 weeks, **b** 28 weeks, and **c** 52 weeks of age. Kidneys of deficiency (*Df(13)Ms2Dja* ^*+/−*^) mice showed mild through moderate to severe dilation of the pelvicalyceal system (*left to right*) (visible as *empty spaces*; indicated by *asterisks*) compared with control littermates (normal structure of the pelvicalyceal system indicated by *asterisks*). Images are representative and taken at ×250 magnification

To determine whether histopathological abnormalities of Sotos patients, in particular, advanced bone age, cardiovascular, and/or urinary/renal abnormalities, could be observed in *Df(13)Ms2Dja* ^*+/−*^ mice, they were subjected to histopathological analysis at 10 weeks of age. Examination of a variety of tissues stained with haematoxylin and eosin (H&E) failed to reveal the presence of anatomical abnormalities of any organs, except the kidneys, where dilation of the pelvicalyceal system was observed in *Df(13)Ms2Dja* ^*+/−*^ mice with 100 % penetrance (Fig. 4a). Kidney and urinary abnormalities are diagnosed in approximately 10 % of Sotos patients, suggesting that kidney abnormalities are a highly penetrant trait recapitulated in *Df(13)Ms2Dja* ^*+/−*^ mice. Interestingly, a range in the severity of the pelvicalyceal system dilatation (mild through moderate to severe) was observed, and in all cases of moderate or severe dilation, only one kidney was affected moderately or severely while the other kidney showed only mild dilation. This dilation did not progress with age, as the same range of changes observed in 10-week-old mice was also present in 28-week-old (Fig. 4b) and 52-week-old (Fig. 4c) deficiency mice. Blood chemistry analysis revealed no significant change in parameters related to urinary/renal system clearance and metabolic functioning, including creatinine, urea, and electrolytes in 28- and 52-week-old *Df(13)Ms2Dja* ^*+/−*^ mice when compared to wild-type littermate controls (data not shown). This suggests that dilation of the pelvicalyceal system does not affect overall kidney function. X-ray analysis at 28 and 52 weeks of age revealed the absence of kidney stones in *Df(13)Ms2Dja* ^*+/−*^ mice (data not shown).

## Discussion

We generated and phenotypically characterized the heterozygous deficiency *Df(13)Ms2Dja* mouse—a genetic model of Sotos syndrome covering a 1.5-Mb region syntenic to the telomeric part of human chromosome 5. This region contains 36 genes, which are conserved between human and mouse (Fig. 1). *Df(13)Ms2Dja* ^*+/−*^ mice were viable, fertile, and indistinguishable from wild-type littermate controls in terms of head and facial morphology, muscle tone, and curvature of their spine. Thus, *Df(13)Ms2Dja* ^*+/−*^ mice do not recapitulate the macrocephaly, facial abnormalities, hypotonia, or scoliosis observed in some Sotos patients (Tatton-Brown and Rahman 2007). However, it is difficult to reliably model facial abnormalities in mice (Tobin et al. 2008). Sotos individuals have facial abnormalities that include a high and broad forehead (the head is said to resemble an inverted pear), frontotemporal hair sparsity, malar flushing, down-slanting palpebral fissures, and a pointed chin, dysmorphology that is probably hard to observe in mice.

Deficiency mice fed on a normal-fat diet did, however, show significantly reduced growth at all time points measured (embryonic day 15.5–52 weeks of age; Fig. 2). The presence of reduced postnatal growth in *Df(13)Ms2Dja* ^*+/−*^ mice contrasts with human data, as Sotos patients with intragenic *NSD1* mutations and those with 5q35 microdeletions encompassing the *NSD1* gene exhibit overgrowth (Tatton-Brown and Rahman 2007). However, the overgrowth phenotype observed in Sotos patients with 5q35 microdeletions is not very pronounced, even at the early stages of development (Kurotaki et al. 2003; Nagai et al. 2003; Saugier-Veber et al. 2007; Tatton-Brown and Rahman 2007). Collectively, these data suggest divergent roles for *Nsd1/NSD1* in regulating growth in mouse and man.

*Df(13)Ms2Dja* ^*+/−*^ mice showed deficits in long-term memory retention in a socially relevant testing paradigm (Fig. 3). In contrast to other artificial novel object discrimination assays, the mechanism of long-term social recognition in mice is multisensory, requiring the integration of both volatile and nonvolatile olfactory and pheromone cues from conspecifics (Noack et al. 2010). Moreover, the sensitivity of the assay is enhanced by its ethological relevance; it does not rely on prior conditioning and instead exploits an innate olfactory-mediated behavioural response of rodents (Engelmann et al. 2011). Social recognition is hippocampus-dependent (Kogan et al. 2000) and is well established for learning and memory testing (Engelmann et al. 2011; Richter et al. 2005) and for assessing cognitive impairment in mice (Mitsui et al. 2009). Thus, *Df(13)Ms2Dja* ^*+/−*^ mice may potentially model the intellectual/learning disability observed in Sotos syndrome patients (Tatton-Brown et al. 2005b). Given that the *4732471D19Rik*-*B4galt7* region spans 36 genes, it is possible that other genes within the interval might, in addition to *Nsd1*, contribute to the cognitive phenotype observed in *Df(13)Ms2Dja* ^*+/−*^ mice.

Histopathological examination of tissues from *Df(13)Ms2Dja* ^*+/-*^ mice revealed no signs of advanced bone age or cardiovascular anomalies, although these abnormalities are observed only in a subset of Sotos patients carrying 5q35 microdeletions (Nagai et al. 2003; Saugier-Veber et al. 2007; Tatton-Brown and Rahman 2007). However, kidney abnormalities were observed in our deficiency animals, specifically dilation of the pelvicalyceal system (Fig. 4). Anomalies of the urinary/renal system, including vesicoureteric reflux, hydronephrosis, and small kidneys, are diagnosed in some (~10 %) Sotos patients carrying 5q35 microdeletions (Kurotaki et al. 2003; Nagai et al. 2003; Saugier-Veber et al. 2007; Tatton-Brown and Rahman 2007). Hydronephrosis is characterized by dilation of the renal pelvis and calyces, so *Df(13)Ms2Dja* ^*+/−*^ mice seem to recapitulate key features of this anomaly. To date, no mice carrying knockout mutations in genes mapped within the 5q35 interval have been shown to exhibit dilation of the pelvicalyceal system, suggesting that *Sncb*, *Unc5a*, *Nsd1*, *Fgfr4*, *Mxd3*, *Rgs14*, *Slc34a1*, *F12*, *Grk6*, *Dbn1*, and *Dok3* are unlikely, at least individually, to contribute to this phenotype in *Df(13)Ms2Dja* ^*+/−*^ mice (Mouse Genome Informatics, http://www.informatics.jax.org). However, it is possible that haploinsufficiency of other genes in the interval may contribute with *Nsd1* to the renal organ system anomalies observed in Sotos patients and *Df(13)Ms2Dja* ^*+/−*^ mice. Synergy between genes within copy number variable regions has recently been demonstrated for cardiac development anomalies associated with Down syndrome (Grossman et al. 2011) and postulated for other genomic disorders (Lupski et al. 2011).

We show here that haploinsufficiency of gene(s) in the *4732471D19Rik-B4galt7* region of MMU13, syntenic to human 5q35.2–q35.3, results in pre- and postnatal undergrowth, cognitive impairment, and dilation of the renal pelvicalyceal system in *Df(13)Ms2Dja* ^*+/−*^ mice. Thus, *Df(13)Ms2Dja* ^*+/−*^ mice have contributed new insights into the role that genes within the Sotos syndrome deletion interval play in Sotos-associated intellectual disability, growth, and development of the urinary/renal system.

## Electronic supplementary material

Below is the link to the electronic supplementary material.

Supplementary material 1 (DOC 128 kb)

Supplementary Fig. 1 Analysis of E15.5 embryo body weight. Body weight measurements in control (*+/+*) and deficiency (*Df(13)Ms2Dja* ^*+/-*^) littermates at embryonic day 15.5. **A** Control (*n* = 15) and deficiency (*Df(13)Ms2Dja* ^*+/-*^, *n* = 10) littermates backcrossed one generation to BALB/cJ. **B** Control (*n* = 13) and deficiency (*Df(13)Ms2Dja* ^*+/-*^, *n* = 13) littermates backcrossed one generation to CBA/J. **C** Control (*n* = 9) and deficiency (*Df(13)Ms2Dja* ^*+/-*^; *n* = 7) littermates backcrossed one generation to 129/S5SvEv. Data were statistically analysed using the two-tailed Student’s *t*-test. Asterisk indicates statistical significance and error bars represent the standard deviation of the measurements (EPS 335 kb)

Supplementary Fig. 2 Olfactory function test. Both deficiency (*Df(13)Ms2Dja* ^*+/-*^, *n* = 5) and wild-type (*+/+*, *n* = 7) animals preferentially investigated novel odours when given a choice between an odourised stimulus [female odours (FO)] and a control (clean cage) in sequential trials. Deficiency and wild-type animals were able to distinguish the novel FO (two-way ANOVA factor with repeated measures for odour, *P* < 0.05); the error bars represent the standard error of the mean). There was no effect of genotype (*P* = 0.2229), suggesting that deficiency mice are not deficient in detecting socially relevant (FO) odours. (EPS 118 kb)

## References

[CR1] Adams DJ, Biggs PJ, Cox T, Davies R, van der Weyden L, Jonkers J, Smith J, Plumb B, Taylor R, Nishijima I, Yu Y, Rogers J, Bradley A (2004). Mutagenic insertion and chromosome engineering resource (MICER). Nat Genet.

[CR2] Albers CA, Paul DS, Schulze H, Freson K, Stephens JC, Smethurst PA, Jolley JD, Cvejic A, Kostadima M, Bertone P, Breuning MH, Debili N, Deloukas P, Favier R, Fiedler J, Hobbs CM, Huang N, Hurles ME, Kiddle G, Krapels I, Nurden P, Ruivenkamp CA, Sambrook JG, Smith K, Stemple DL, Strauss G, Thys C, van Geet C, Newbury-Ecob R, Ouwehand WH, Ghevaert C (2012). Compound inheritance of a low-frequency regulatory SNP and a rare null mutation in exon-junction complex subunit RBM8A causes TAR syndrome. Nat Genet.

[CR3] Baujat G, Rio M, Rossignol S, Sanlaville D, Lyonnet S, Le Merrer M, Munnich A, Gicquel C, Cormier-Daire V, Colleaux L (2004). Paradoxical NSD1 mutations in Beckwith-Wiedemann syndrome and 11p15 anomalies in Sotos syndrome. Am J Hum Genet.

[CR4] Engelmann M, Hadicke J, Noack J (2011). Testing declarative memory in laboratory rats and mice using the nonconditioned social discrimination procedure. Nat Protoc.

[CR5] Grossman TR, Gamliel A, Wessells RJ, Taghli-Lamallem O, Jepsen K, Ocorr K, Korenberg JR, Peterson KL, Rosenfeld MG, Bodmer R, Bier E (2011). Over-expression of DSCAM and COL6A2 cooperatively generates congenital heart defects. PLoS Genet.

[CR6] Kogan JH, Frankland PW, Silva AJ (2000). Long-term memory underlying hippocampus-dependent social recognition in mice. Hippocampus.

[CR7] Kurotaki N, Harada N, Yoshiura K, Sugano S, Niikawa N, Matsumoto N (2001). Molecular characterization of NSD1, a human homologue of the mouse *Nsd1* gene. Gene.

[CR8] Kurotaki N, Imaizumi K, Harada N, Masuno M, Kondoh T, Nagai T, Ohashi H, Naritomi K, Tsukahara M, Makita Y, Sugimoto T, Sonoda T, Hasegawa T, Chinen Y, Tomita Ha HA, Kinoshita A, Mizuguchi T, Yoshiura Ki K, Ohta T, Kishino T, Fukushima Y, Niikawa N, Matsumoto N (2002). Haploinsufficiency of NSD1 causes Sotos syndrome. Nat Genet.

[CR9] Kurotaki N, Harada N, Shimokawa O, Miyake N, Kawame H, Uetake K, Makita Y, Kondoh T, Ogata T, Hasegawa T, Nagai T, Ozaki T, Touyama M, Shenhav R, Ohashi H, Medne L, Shiihara T, Ohtsu S, Kato Z, Okamoto N, Nishimoto J, Lev D, Miyoshi Y, Ishikiriyama S, Sonoda T, Sakazume S, Fukushima Y, Kurosawa K, Cheng JF, Yoshiura K, Ohta T, Kishino T, Niikawa N, Matsumoto N (2003). Fifty microdeletions among 112 cases of Sotos syndrome: low copy repeats possibly mediate the common deletion. Hum Mutat.

[CR10] Kurotaki N, Shen JJ, Touyama M, Kondoh T, Visser R, Ozaki T, Nishimoto J, Shiihara T, Uetake K, Makita Y, Harada N, Raskin S, Brown CW, Hoglund P, Okamoto N, Lupski JR (2005). Phenotypic consequences of genetic variation at hemizygous alleles: Sotos syndrome is a contiguous gene syndrome incorporating coagulation factor twelve (FXII) deficiency. Genet Med.

[CR11] Li M, Shuman C, Fei YL, Cutiongco E, Bender HA, Stevens C, Wilkins-Haug L, Day-Salvatore D, Yong SL, Geraghty MT, Squire J, Weksberg R (2001). GPC3 mutation analysis in a spectrum of patients with overgrowth expands the phenotype of Simpson-Golabi-Behmel syndrome. Am J Med Genet.

[CR12] Lupski JR, Belmont JW, Boerwinkle E, Gibbs RA (2011). Clan genomics and the complex architecture of human disease. Cell.

[CR13] Migdalska AM, van der Weyden L, Ismail O, White JK, Sanchez-Andrade G, Logan DW, Arends MJ, Adams DJ (2012). Modeling partial monosomy for human chromosome 21q11.2–q21.1 reveals haploinsufficient genes influencing behavior and fat deposition. PLoS One.

[CR14] Mitsui S, Osako Y, Yokoi F, Dang MT, Yuri K, Li Y, Yamaguchi N (2009). A mental retardation gene, motopsin/neurotrypsin/prss12, modulates hippocampal function and social interaction. Eur J Neurosci.

[CR15] Nagai T, Matsumoto N, Kurotaki N, Harada N, Niikawa N, Ogata T, Imaizumi K, Kurosawa K, Kondoh T, Ohashi H, Tsukahara M, Makita Y, Sugimoto T, Sonoda T, Yokoyama T, Uetake K, Sakazume S, Fukushima Y, Naritomi K (2003). Sotos syndrome and haploinsufficiency of NSD1: clinical features of intragenic mutations and submicroscopic deletions. J Med Genet.

[CR16] Noack J, Richter K, Laube G, Haghgoo HA, Veh RW, Engelmann M (2010). Different importance of the volatile and non-volatile fractions of an olfactory signature for individual social recognition in rats versus mice and short-term versus long-term memory. Neurobiol Learn Mem.

[CR17] Ramirez-Solis R, Liu P, Bradley A (1995). Chromosome engineering in mice. Nature.

[CR18] Rayasam GV, Wendling O, Angrand PO, Mark M, Niederreither K, Song L, Lerouge T, Hager GL, Chambon P, Losson R (2003). NSD1 is essential for early post-implantation development and has a catalytically active SET domain. EMBO J.

[CR19] Richter K, Wolf G, Engelmann M (2005). Social recognition memory requires two stages of protein synthesis in mice. Learn Mem.

[CR20] Robertson E, Robertson E (1987). Embryo-derived stem cell lines. Teratocarcinomas and embryonic stem cells: a practical approach.

[CR21] Saugier-Veber P, Bonnet C, Afenjar A, Drouin-Garraud V, Coubes C, Fehrenbach S, Holder-Espinasse M, Roume J, Malan V, Portnoi MF, Jeanne N, Baumann C, Heron D, David A, Gerard M, Bonneau D, Lacombe D, Cormier-Daire V, Billette de Villemeur T, Frebourg T, Burglen L (2007). Heterogeneity of NSD1 alterations in 116 patients with Sotos syndrome. Hum Mutat.

[CR22] Su H, Mills AA, Wang X, Bradley A (2002). A targeted X-linked CMV-Cre line. Genesis.

[CR23] Tatton-Brown K, Rahman N (2007). Sotos syndrome. Eur J Hum Genet.

[CR24] Tatton-Brown K, Cole TRP, Rahman N, Pagon RA, Bird TD, Dolan CR, Stephens K, Adam MP (1993). Sotos syndrome. GeneReviews.

[CR25] Tatton-Brown K, Douglas J, Coleman K, Baujat G, Chandler K, Clarke A, Collins A, Davies S, Faravelli F, Firth H, Garrett C, Hughes H, Kerr B, Liebelt J, Reardon W, Schaefer GB, Splitt M, Temple IK, Waggoner D, Weaver DD, Wilson L, Cole T, Cormier-Daire V, Irrthum A, Rahman N (2005). Multiple mechanisms are implicated in the generation of 5q35 microdeletions in Sotos syndrome. J Med Genet.

[CR26] Tatton-Brown K, Douglas J, Coleman K, Baujat G, Cole TR, Das S, Horn D, Hughes HE, Temple IK, Faravelli F, Waggoner D, Türkmen S, Cormier-Daire V, Irrthum A, Rahman N, The Childhood Overgrowth Collaboration (2005). Genotype-phenotype associations in Sotos syndrome: an analysis of 266 individuals with NSD1 aberrations. Am J Hum Genet.

[CR27] Tobin JL, Di Franco M, Eichers E, May-Simera H, Garcia M, Yan J, Quinlan R, Justice MJ, Hennekam RC, Briscoe J, Tada M, Mayor R, Burns AJ, Lupski JR, Hammond P, Beales PL (2008). Inhibition of neural crest migration underlies craniofacial dysmorphology and Hirschsprung’s disease in Bardet–Biedl syndrome. Proc Natl Acad Sci USA.

[CR28] Zheng B, Mills AA, Bradley A (1999). A system for rapid generation of coat color-tagged knockouts and defined chromosomal rearrangements in mice. Nucleic Acids Res.

