# Supplementary material for: Generation of the Sotos syndrome deletion in mice
Source: Mamm Genome. 2012 Aug 29;23(11):749–57. doi: 10.1007/s00335-012-9416-0 (PMC3510424; doi:10.1007/s00335-012-9416-0)
Supplement: Supplementary file 1 — Supplementary material 1 (DOC 128 kb) [file 335_2012_9416_MOESM1_ESM.doc]

**Table S1.** **Summary of the genes mapped within the *4732471D19Rik−B4galt7* region (NCBI build m37).** Functions/processes were gene-based on ontology classifications as listed by Mouse Genome Informatics (<http://www.informatics.jax.org/>).

| **Gene** | **Official name** | **Ensembl ID (v61)** | **Function/Processes (GO terms)** |
| --- | --- | --- | --- |
| [*4732471D19Rik*](http://www.ensembl.org/Mus_musculus/geneview?gene=4732471D19Rik) | RIKEN cDNA 4732471D19 gene | ENSMUSG00000043183 | Unknown |
| [*4833439L19Rik*](http://www.ensembl.org/Mus_musculus/geneview?gene=4833439L19Rik) | RIKEN cDNA 4833439L19 gene | ENSMUSG00000025871 | Unknown |
| *Arl10* | ADP-ribosylation factor-like 10 | [ENSMUSG00000025870](http://www.ensembl.org/Mus_musculus/geneview?gene=ENSMUSG00000025870) | GTP and nucleotide binding activity |
| *Nop16* | NOP16 nucleolar protein homolog (yeast) | [ENSMUSG00000025869](http://www.ensembl.org/Mus_musculus/geneview?gene=ENSMUSG00000025869) | Unknown |
| [*Higd2a*](http://www.ensembl.org/Mus_musculus/geneview?gene=Higd2a) | HIG1 domain family, member 2A | [ENSMUSG00000025868](http://www.ensembl.org/Mus_musculus/geneview?gene=ENSMUSG00000025868) | Unknown |
| [*Cltb*](http://www.ensembl.org/Mus_musculus/geneview?gene=Cltb) | Clathrin, light polypeptide (Lcb) | ENSMUSG00000045547 | Intracellular protein transport, vesicle-mediated transport, and peptide binding activity |
| [*Faf2*](http://www.ensembl.org/Mus_musculus/geneview?gene=Faf2) | Fas associated factor family member 2 | [ENSMUSG00000025873](http://www.ensembl.org/Mus_musculus/geneview?gene=ENSMUSG00000025873) | Response to unfolded proteins, and ubiquitin and ubiquitin protein ligase binding |
| [*Rnf44*](http://www.ensembl.org/Mus_musculus/geneview?gene=Rnf44) | Ring finger protein 44 | [ENSMUSG00000034928](http://www.ensembl.org/Mus_musculus/geneview?gene=ENSMUSG00000034928) | Metal ion binding activity |
| *Cdhr2* | Cadherin-related family member 2 | ENSMUSG00000034918 | Cell adhesion, and negative regulation of cell growth |
| *Gprin1* | G protein-regulated inducer of neurite outgrowth 1 | [ENSMUSG00000069227](http://www.ensembl.org/Mus_musculus/geneview?gene=ENSMUSG00000069227) | Neuron projection development, and phospoprotein binding activity |
| *Sncb* | Synuclein, beta | [ENSMUSG00000034891](http://www.ensembl.org/Mus_musculus/geneview?gene=ENSMUSG00000034891) | Dopamine metabolic processes, negative regulation of neuron apoptosis, and calcium ion binding activity |
| [*Eif4e1b*](http://www.ensembl.org/Mus_musculus/geneview?gene=Eif4e1b) | Eukaryotic translation initiation factor 4E family member 1B | [ENSMUSG00000074895](http://www.ensembl.org/Mus_musculus/geneview?gene=ENSMUSG00000074895) | Translational, and RNA binding and translation initiation factor activity |
| [*Tspan17*](http://www.ensembl.org/Mus_musculus/geneview?gene=Tspan17) | Tetraspanin 17 | [ENSMUSG00000025875](http://www.ensembl.org/Mus_musculus/geneview?gene=ENSMUSG00000025875) | Unknown |
| [*Unc5a*](http://www.ensembl.org/Mus_musculus/geneview?gene=Unc5a) | Unc-5 homolog A (*C.elegans*) | [ENSMUSG00000025876](http://www.ensembl.org/Mus_musculus/geneview?gene=ENSMUSG00000025876) | Apoptosis, axon guidance, multicellular organismal development, and netrin receptor activity |
| [*Hk3*](http://www.ensembl.org/Mus_musculus/geneview?gene=Hk3) | Hexokinase 3 | [ENSMUSG00000025877](http://www.ensembl.org/Mus_musculus/geneview?gene=ENSMUSG00000025877) | Carbohydrate metabolic processes, phosphorylation, glycolysis, kinase and transferase activity, and ATP, kinase, hormone, enzyme and hexokinase binding activity |
| [*Uimc1*](http://www.ensembl.org/Mus_musculus/geneview?gene=Uimc1) | Ubiquitin interaction motif containing 1 | [ENSMUSG00000025878](http://www.ensembl.org/Mus_musculus/geneview?gene=ENSMUSG00000025878) | Chromatin modifications, DNA repair, DNA-dependent, negative regulation of transcription, [histone H2A K63-linked deubiquitination](http://www.informatics.jax.org/searches/GO.cgi?id=GO:0070537), and histone and [K63-linked polyubiquitin binding](http://www.informatics.jax.org/searches/GO.cgi?id=GO:0070530) activity |
| [*Zfp346*](http://www.ensembl.org/Mus_musculus/geneview?gene=Zfp346) | Zinc finger protein 346 | [ENSMUSG00000021481](http://www.ensembl.org/Mus_musculus/geneview?gene=ENSMUSG00000021481) | Apoptosis, and metal ion and double-stranded RNA binding activity |
| [*Fgfr4*](http://www.ensembl.org/Mus_musculus/geneview?gene=Fgfr4) | Fibroblast growth factor receptor 4 | [ENSMUSG00000005320](http://www.ensembl.org/Mus_musculus/geneview?gene=ENSMUSG00000005320) | [Alveolar secondary septum development](http://www.informatics.jax.org/searches/GO.cgi?id=GO:0061144), [fibroblast growth factor receptor signalling pathway](http://www.informatics.jax.org/searches/GO.cgi?id=GO:0008543), protein phosphorylation, organ induction, lung development, and ATP, kinase and transferase binding activity |
| [*Nsd1*](http://www.ensembl.org/Mus_musculus/geneview?gene=Nsd1) | Nuclear receptor-binding SET-domain protein 1 | [ENSMUSG00000021488](http://www.ensembl.org/Mus_musculus/geneview?gene=ENSMUSG00000021488) | Gastrulation with mouth forming second, histone H3-K36 methylation, and androgen receptor, zinc ion, transcription cofactor and chromatin binding activity |
| [*Rab24*](http://www.ensembl.org/Mus_musculus/geneview?gene=Rab24) | RAB24, member RAS oncogene family | [ENSMUSG00000034789](http://www.ensembl.org/Mus_musculus/geneview?gene=ENSMUSG00000034789) | Autophagy, protein transport, small GTPase mediated signal transduction, and GTP and nucleotide binding activity |
| [*Mxd3*](http://www.ensembl.org/Mus_musculus/geneview?gene=Mxd3) | Max dimerization protein 3 | [ENSMUSG00000021485](http://www.ensembl.org/Mus_musculus/geneview?gene=ENSMUSG00000021485) | [Negative regulation of transcription, DNA-dependent](http://www.informatics.jax.org/searches/GO.cgi?id=GO:0045892) regulation of transcription, and protein binding activity |
| [*Prelid1*](http://www.ensembl.org/Mus_musculus/geneview?gene=Prelid1) | PRELI domain containing 1 | [ENSMUSG00000021486](http://www.ensembl.org/Mus_musculus/geneview?gene=ENSMUSG00000021486) | Unknown |
| [*Lman2*](http://www.ensembl.org/Mus_musculus/geneview?gene=Lman2) | Lectin, mannose-binding 2 | [ENSMUSG00000021484](http://www.ensembl.org/Mus_musculus/geneview?gene=ENSMUSG00000021484) | Protein transport, and metal ion and sugar binding |
| [*Rgs14*](http://www.ensembl.org/Mus_musculus/geneview?gene=Rgs14) | Regulator of G-protein signalling 14 | [ENSMUSG00000052087](http://www.ensembl.org/Mus_musculus/geneview?gene=ENSMUSG00000052087) | Cell cycle and division, [spindle organization](http://www.informatics.jax.org/searches/GO.cgi?id=GO:0007051), [cell division](http://www.informatics.jax.org/searches/GO.cgi?id=GO:0051301), and [GDP dissociation inhibitor,](http://www.informatics.jax.org/searches/GO.cgi?id=GO:0005092) [microtubule binding](http://www.informatics.jax.org/searches/GO.cgi?id=GO:0008017) activity, and [GTPase activator activity](http://www.informatics.jax.org/searches/GO.cgi?id=GO:0005096), and [positive regulation of GTPase activity](http://www.informatics.jax.org/searches/GO.cgi?id=GO:0043547) |
| [*Slc34a1*](http://www.ensembl.org/Mus_musculus/geneview?gene=Slc34a1) | Solute carrier family 34 (sodium phosphate), member 1 | [ENSMUSG00000021490](http://www.ensembl.org/Mus_musculus/geneview?gene=ENSMUSG00000021490) | Ion transport, bone remodelling, and [sodium-dependent phosphate transmembrane transporter activity](http://www.informatics.jax.org/searches/GO.cgi?id=GO:0015321) |
| [*Pfn3*](http://www.ensembl.org/Mus_musculus/geneview?gene=Pfn3) | Profilin 3 | [ENSMUSG00000044444](http://www.ensembl.org/Mus_musculus/geneview?gene=ENSMUSG00000044444) | Actin cytoskeleton organization, and [actin binding](http://www.informatics.jax.org/searches/GO.cgi?id=GO:0003779) and [ATP:ADP antiporter activity](http://www.informatics.jax.org/searches/GO.cgi?id=GO:0005471) |
| [*F12*](http://www.ensembl.org/Mus_musculus/geneview?gene=F12) | Coagulation factor XII (Hageman factor) | [ENSMUSG00000021492](http://www.ensembl.org/Mus_musculus/geneview?gene=ENSMUSG00000021492) | [Plasma kallikrein-kinin cascade](http://www.informatics.jax.org/searches/GO.cgi?id=GO:0002353), [Factor XII activation](http://www.informatics.jax.org/searches/GO.cgi?id=GO:0002542), [positive regulation of plasminogen activation](http://www.informatics.jax.org/searches/GO.cgi?id=GO:0010756), [protein autoprocessing](http://www.informatics.jax.org/searches/GO.cgi?id=GO:0016540), [positive regulation of blood coagulation](http://www.informatics.jax.org/searches/GO.cgi?id=GO:0030194), [zymogen activation](http://www.informatics.jax.org/searches/GO.cgi?id=GO:0031638), [protein maturation by peptide bond cleavage](http://www.informatics.jax.org/searches/GO.cgi?id=GO:0051605), [response to misfolded protein](http://www.informatics.jax.org/searches/GO.cgi?id=GO:0051788), and [positive regulation of fibrinolysis](http://www.informatics.jax.org/searches/GO.cgi?id=GO:0051919) |
| [*Grk6*](http://www.ensembl.org/Mus_musculus/geneview?gene=Grk6) | G protein-coupled receptor kinase 6 | [ENSMUSG00000074886](http://www.ensembl.org/Mus_musculus/geneview?gene=ENSMUSG00000074886) | Desensitization of G-protein coupled receptor protein signalling pathway, protein phosphorylation, and ATP binding, and transferase and protein serine/threonine kinase activity |
| [*Prr7*](http://www.ensembl.org/Mus_musculus/geneview?gene=Prr7) | Proline rich 7 (synaptic) | [ENSMUSG00000034686](http://www.ensembl.org/Mus_musculus/geneview?gene=ENSMUSG00000034686) | Unknown |
| [*Dbn1*](http://www.ensembl.org/Mus_musculus/geneview?gene=Dbn1) | Drebrin 1 | [ENSMUSG00000034675](http://www.ensembl.org/Mus_musculus/geneview?gene=ENSMUSG00000034675) | Actin filament organization, signal transduction, multicellular organismal development, nervous system development, cell differentiation, and actin and profiling binding activity |
| [*Pdlim7*](http://www.ensembl.org/Mus_musculus/geneview?gene=Pdlim7) | PDZ and LIM domain 7 | [ENSMUSG00000021493](http://www.ensembl.org/Mus_musculus/geneview?gene=ENSMUSG00000021493) | Actin cytoskeleton organization, ossification, multicellular organismal development, cell differentiation, and metal ion binding activity |
| [*Dok3*](http://www.ensembl.org/Mus_musculus/geneview?gene=Dok3) | Docking protein 3 | [ENSMUSG00000035711](http://www.ensembl.org/Mus_musculus/geneview?gene=ENSMUSG00000035711) | Ras protein signal transduction, and insulin receptor binding activity |
| *Ddx41* | DEAD (Asp-Glu-Ala-Asp) box polypeptide 41 | [ENSMUSG00000021494](http://www.ensembl.org/Mus_musculus/geneview?gene=ENSMUSG00000021494) | mRNA processing, RNA splicing, and [metal ion binding](http://www.informatics.jax.org/searches/GO.cgi?id=GO:0046872), [ATP binding](http://www.informatics.jax.org/searches/GO.cgi?id=GO:0005524) and [helicase activity](http://www.informatics.jax.org/searches/GO.cgi?id=GO:0004386) |
| [*Fam193b*](http://www.ensembl.org/Mus_musculus/geneview?gene=BC021381) | Family with sequence similarity 193, member B | [ENSMUSG00000021495](http://www.ensembl.org/Mus_musculus/geneview?gene=ENSMUSG00000021495) | Unknown |
| [*Tmed9*](http://www.ensembl.org/Mus_musculus/geneview?gene=Tmed9) | Transmembrane emp24 protein transport domain containing 9 | [ENSMUSG00000058569](http://www.ensembl.org/Mus_musculus/geneview?gene=ENSMUSG00000058569) | Transport |
| [*B4galt7*](http://www.ensembl.org/Mus_musculus/geneview?gene=B4galt7) | Xylosylprotein beta1,4-galactosyltransferase, polypeptide 7 (galactosyltransferase I) | [ENSMUSG00000021504](http://www.ensembl.org/Mus_musculus/geneview?gene=ENSMUSG00000021504) | Carbohydrate and proteoglycan metabolic process, glycosaminoglycan biosynthetic process, fibril organization, negative regulation of fibroblast proliferation, and metal ion binding, [transferase and transferring glycosyl groups](http://www.informatics.jax.org/searches/GO.cgi?id=GO:0016757) activity |
